# Simplified Assessment of the Index of Microvascular Resistance

**DOI:** 10.1155/2021/9971874

**Published:** 2021-06-02

**Authors:** Monika Kodeboina, Sakura Nagumo, Daniel Munhoz, Jeroen Sonck, Niya Mileva, Emanuele Gallinoro, Alessandro Candreva, Takuya Mizukami, Frederik Van Durme, Alex Heyse, Eric Wyffels, Marc Vanderheyden, Emanuele Barbato, Jozef Bartunek, Bernard De Bruyne, Carlos Collet

**Affiliations:** ^1^Cardiovascular Center Aalst, OLV Clinic, Aalst, Belgium; ^2^Department of Advanced Biomedical Sciences, University Federico II, Naples, Italy; ^3^Division of Cardiology, Department of Internal Medicine, Showa University Fujigaoka Hospital, Yokohama, Kanagawa, Japan; ^4^Department of Internal Medicine, Discipline of Cardiology, University of Campinas (Unicamp), Campinas, Brazil; ^5^Cardiology Clinic Alexandrovska University Hospital, Sofia, Bulgaria; ^6^Department of Translational Medical Sciences, University of Campania “Luigi Vanvitelli”, Naples, Italy; ^7^Department of Interventional Cardiology, University Heart Center, University Hospital Zurich, Zurich, Switzerland; ^8^Department of Pharmacology, Showa University School of Medicine, Tokyo, Japan; ^9^Department of Cardiology, Lausanne University Hospital, Lausanne, Switzerland

## Abstract

**Background:**

To validate a simplified invasive method for the calculation of the index of microvascular resistance (IMR).

**Methods:**

This is a prospective, single-center study of patients with chronic coronary syndromes presenting with nonobstructive coronary artery disease. IMR was obtained using both intravenous (IV) adenosine and intracoronary (IC) papaverine. Each IMR measurement was obtained in duplicate. The primary objective was the agreement between IMR acquired using adenosine and papaverine. Secondary objectives include reproducibility of IMR and time required for the IMR measurement.

**Results:**

One hundred and sixteen IMR measurements were performed in 29 patients. The mean age was 68.8 ± 7.24 years, and 27.6% was diabetics. IMR values were similar between papaverine and adenosine (17.7 ± 7.26 and 20.1 ± 8.6, *p*=0.25; Passing-Bablok coefficient A 0.58, 95% CI −2.42 to 3.53; coefficient B 0.90, 95% CI −0.74 to 1.07). The reproducibility of IMR was excellent with both adenosine and papaverine (ICC 0.78, 95% CI 0.63 to 0.88 and ICC 0.93, 95% CI 0.87 to 0.97). The time needed for microvascular assessment was significantly shortened by the use of IC papaverine (3.23 (2.84, 3.78) mins vs. 5.48 (4.94, 7.09) mins, *p* < 0.0001).

**Conclusion:**

IMR can be reliably measured using IC papaverine with similar results compared to intravenous infusion of adenosine with increased reproducibility and reduced procedural time. This approach simplifies the invasive assessment of the coronary microcirculation in the catheterization laboratory.

## 1. Introduction

Coronary microvascular dysfunction (CMD) remains an ill-defined condition. Accordingly, guidelines recommend the invasive assessment of the coronary microvascular resistance in patients with angina and nonobstructive coronary artery disease [1]. CMD has a crucial role modulating patient outcomes in various clinical settings, including acute coronary syndromes, after percutaneous coronary interventions, in patients with cardiomyopathies and cardiac transplantation-related allograft vasculopathy [2–5]. Furthermore, microvascular angina negatively impacts the quality of life [6–8].

The IMR technique is based on bolus thermodilution-derived mean transit time [9, 10] during pharmacologically induced hyperemia [11]. An IMR greater than 25 units is considered to be abnormal and diagnostic criterion for microvascular angina [12]. IMR has emerged as an invasive reproducible method for assessing the coronary microcirculation [13]. In the catheterization laboratory, intravenous (IV) adenosine has been the favored hyperemic agent. Nonetheless, this requires prolonged infusions, often leading to patient discomfort and eventually needs a central vein infusion to achieve reliable and stable adenosine hyperemic response [14, 15]. Papaverine is an alternative hyperemic agent suitable for intracoronary administration that induces a stable hyperemic state sufficient to evaluate hyperemic mean transit times and IMR [16].

We sought to assess the feasibility, accuracy, and reproducibility of IMR measurement using intracoronary papaverine with IV adenosine as the reference standard.

## 2. Methods

### 2.1. Study Design

This is a prospective, single-center study of patients with suspected coronary artery disease undergoing coronary angiography. Patients with nonobstructive coronary artery disease defined as epicardial diameter stenosis visually assessed as <50% and FFR >0.8 were included. Patients presenting in the context of acute coronary syndrome, with concomitant significant valve disease, with contraindication for adenosine administration were excluded. Patients underwent microvascular evaluation with bolus thermodilution in rest and hyperemia using IV adenosine and IC papaverine, both acquired in duplicated measurements. The study protocol was approved by the institutional ethics committee (Institutional Ethics Committee 2020/013). All patients signed the written informed consent before the procedure.

### 2.2. Study Procedures

A 6F arterial sheath was introduced in the radial or femoral artery. The left anterior descending artery (LAD) was the preferred vessel for the measurements. After administering 200 *μ*g of nitroglycerine, a guidewire equipped with a pressure and temperature sensor (PressureWire X, Abbott, IL, USA) was first equalized with the aortic pressure at the ostium of the coronary artery and subsequently placed in the distal segment of the coronary artery. Next, temperatures of the proximal and distal sensor were zeroed. The ratio between the distal coronary pressure and aortic pressure (Pd/Pa) was recorded. Three room temperature saline injections of 3 ml were performed to calculate resting mean transit time. Next, intracoronary papaverine (12 mg in the left coronary artery and 8 mg in the right coronary artery) was administrated. After hyperemic stable state, three saline injections of 3 ml were performed to acquire the hyperemic mean transit times. This allowed for the calculation of coronary flow reserve (CFR) and IMR. This measurement was repeated after Pd/Pa returned to baseline values. Afterward, IMR was measured again using an intravenous infusion of adenosine at a rate of 140 mcg/min through a peripheral venous line as previously described [11]. A stable hyperemic state was achieved after continuous infusion of IV adenosine for at least two minutes. Three saline injections of 3 ml were repeated after that. For the present analysis, fractional flow reserve and Pd/Pa values are reported from the first measurements of both IC papaverine and IV adenosine. An IMR ≥ 25 was considered abnormal. The measurement time was defined as from the start of the first bolus thermodilution in rest until the completion of the last bolus thermodilution during hyperemia, including time required for the induction of hyperemia but not the time consumed for the preparation of the drugs.

Coronary angiography was acquired using a prespecified protocol, with at least two projections separated by 30°. Angiographies were analyzed using three-dimensional quantitative coronary angiography (CAAS 8.2 Pie Medical Imaging). TIMI frame count was analyzed as previously described [17].

### 2.3. Statistical Analysis

Continuous values are presented as mean ± SD. Values with a nonparametric distribution were represented by the median (interquartile range). Mean values were compared with Student paired and unpaired *t*-tests. Reproducibility was analyzed with the Bland–Altman method and intraclass correlation (ICC). Reliability was assessed by Cronbach' alpha, which was used as a basis to compare two continuous ICC. Agreement on IMR between papaverine and adenosine was analyzed with Passing-Bablok regression. Cohen's kappa was used to assess the agreement on IMR values stratified by the 25 units cutoff. A *p* value of 0.05 was considered significant. Analyses were performed using *R* (R Foundation, Vienna).

## 3. Results

### 3.1. Baseline Clinical Characteristics

Overall, 29 patients were included. The mean age was 68.8 ± 7.24 years, with a prevalence of males of 62.1%. Baseline clinical characteristics are given in Table 1. All patients underwent CFR and IMR evaluation using IC papaverine and IV adenosine. This resulted in 116 measurements (58 IMR measurements with papaverine and 58 IMR measurements with adenosine).

The most prevalent coronary artery evaluated was the LAD in 16 patients (55.2%), followed by the LCX in 7 patients (24.1%) and the RCA in 6 patients (20.7%). Median percent diameter stenosis was 20.0% (14.0, 32.0). The median TIMI frame count in the LAD was 23.8 ± 7.64, 24.6 ± 12.5 in the LCX and 21 ± 3.52 in the RCA. Angiographic characteristics are given in Table 2.

### 3.2. Functional Assessment

**Figure 1:**
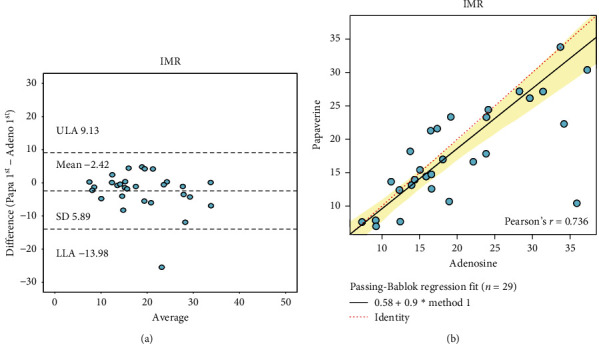
Agreement on IMR between papaverine and adenosine. (a) Bland–Altman plot of IMR measured with papaverine and adenosine. (b) Passing-Bablok regression of IMR measured with papaverine and adenosine.

Mean fractional flow reserve (FFR) was 0.92 ± 0.07 in the papaverine group and 0.90 ± 0.08 in the adenosine group (*p*=0.52). Pa was reduced by both papaverine and adenosine (16.5 ± 8.8 vs. 7.8 ± 10.4 mmHg; *p* < 0.001). IMR was 17.7 ± 7.26 with IC papaverine and 20.1 ± 8.6 with IV adenosine *p*=0.25; Passing-Bablok coefficient A 0.58 (95% CI −2.42 to 3.53), coefficient B 0.90 (95% CI −0.74 to 1.07), as shown in Figure 1. 22.6% of cases showed abnormal IMR with IC papaverine, whereas in 22.6% of the cases, IMR was abnormal with IV adenosine (Cohen's kappa 0.74, 95% CI 0.54 to 0.95).  Table S1 provides the value of each IMR assessment. Pd and hyperemic mean transit time (Tmn) were similar between IC papaverine and IV adenosine (Pd 70.7 ± 15.2 mmHg vs. 69.5 ± 12.5 mmHg; *p*=0.75; Passing-Bablok coefficient A 10.77 (95% CI −13.11 to 27.42); coefficient B 0.84 (95% CI 0.59 to 1.17) and hyperemic Tmn 0.29 ± 0.12 s with papaverine and 0.26 ± 0.11 s with adenosine, *p*=0.33; Passing-Bablok coefficient A −0.01 (95% CI −0.06 to 0.04); coefficient B 0.90 (95% CI 0.78 to 1.14)). Table 2 summarizes the findings of the functional assessment.  Figure S1 provides a comparison between each resting state before a CFR/IMR measurement.

The time required to perform CFR/IMR measurements was significantly reduced using IC papaverine compared to IV adenosine (3.23 (2.84, 3.78) mins with IC papaverine vs. 5.48 (4.94, 7.09) mins with IV adenosine; *p* < 0.0001).

There were no complications observed with the administration of papaverine or adenosine. None of the patients presented any symptoms during the administration of papaverine. During the IV adenosine administration, 31% referred symptoms of shortness of breath and 82% referred chest discomfort.

### 3.3. Assessment of Reproducibility

**Figure 2:**
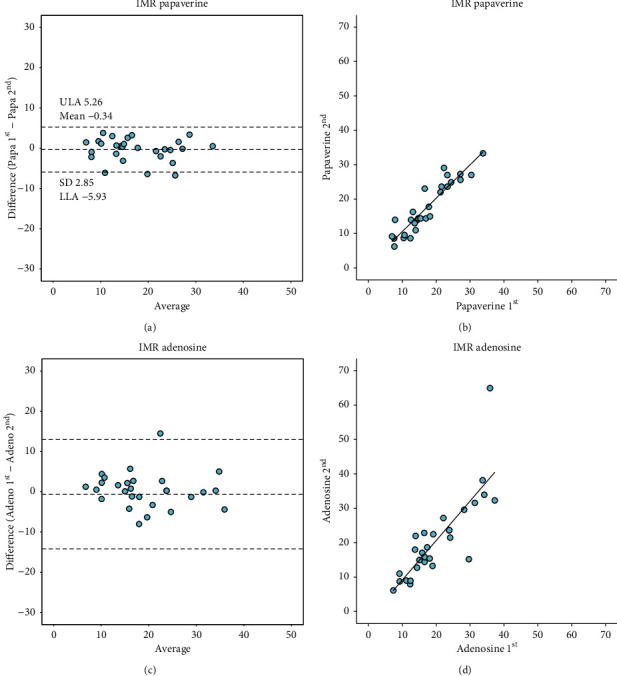
Reproducibility of IMR obtained with IC papaverine and IV adenosine. (a) Bland–Altman plot of IMR with repeated measures of papaverine. (b) Correlation of IMR measured with repeated measures of papaverine. (c) Bland–Altman plot of IMR measured with repeated measures of adenosine. (d) Correlation of IMR measured with repeated measures of adenosine. IMR, index of microvascular resistance; IC, intracoronary; IV, intravenous; Papa 1^st^, first injection of papaverine; Papa 2^nd^, second injection of papaverine; Adeno 1^st^, first injection of adenosine; Adeno 2^nd^, second injection of adenosine.

**Figure 3:**
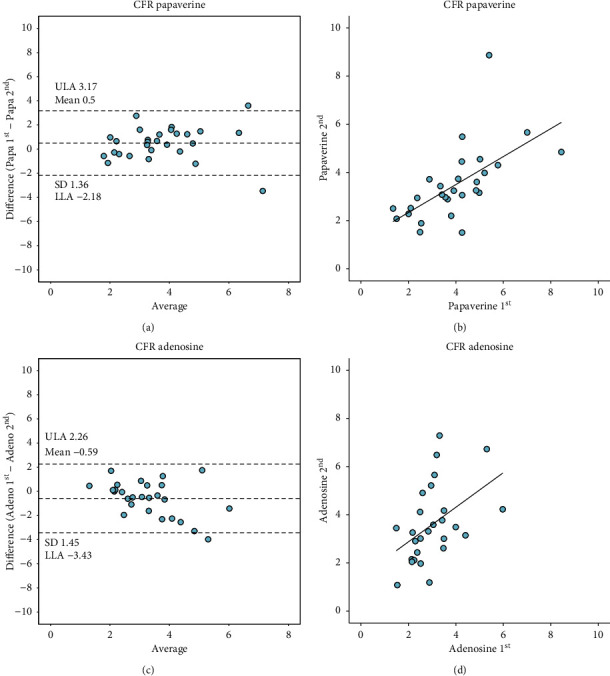
Reproducibility of CFR obtained with IC papaverine and IV adenosine. (a) Bland–Altman plot of CFR with repeated measures of papaverine. (b) Correlation of CFR measured with repeated measures of papaverine. (c) Bland–Altman plot of CFR measured with repeated measures of adenosine. (d) Correlation of CFR measured with repeated measures of adenosine. IMR, index of microvascular resistance; IC, intracoronary; IV, intravenous; Papa 1^st^, first injection of papaverine; Papa 2^nd^, second injection of papaverine; Adeno 1^st^, first injection of adenosine; Adeno 2^nd^, second injection of adenosine.

IMR measurements using IC papaverine showed excellent reproducibility (mean difference −0.34, limits of agreement −5.93 to 5.26, and ICC of 0.93 (95% CI 0.87 to 0.96). Likewise, IMR measurements using IV adenosine showed high reproducibility (mean difference −0.63, limits of agreement −14.2 to 12.95) and ICC of 0.78 (95% CI 0.63 to 0.88).  Figure 2 summarizes IMR reproducibility with both hyperemic agents. The reproducibility of IMR using papaverine was significantly higher compared to the reproducibility of IMR measured using IV adenosine (*p*=0.0156).  Figure 3 summarizes CFR reproducibility. There was a weak correlation between corrected TFC and Tmn (Figures S2 and S3 in the Supplementary Materials).

## 4. Discussion

The main findings of this study can be summarized as follows: (1) IMR with IC papaverine is feasible, (2) IMR using IC papaverine provides similar results to the ones obtained using IV adenosine, (3) IMR measurement using IC papaverine showed higher reproducibility compared to IMR obtained using IV adenosine, and (4) IC papaverine shortened the time required to assess IMR compared to IV adenosine, although drug preparation is not accounted in this time frame.

Approximately half of the patients undergoing coronary angiography, even with a positive noninvasive test, do not exhibit obstructive epicardial coronary artery disease [18]. In these patients, a potential pathophysiological mechanism for myocardial ischemia is CMD. Moreover, identifying CMD as the cause of the patient complaints triggers medical and risk factors management, which has been associated with improvement in angina and quality of life [8]. Nonetheless, a systematic evaluation of the coronary microcirculation in clinical practice is seldom performed. The main limiting factors have been attributed to the necessity of dedicated devices such as pressure/temperature or Doppler wires and the need to induce hyperemia. In the catheterization laboratory, the administration of IV adenosine is time-consuming, costly, and is associated with patient discomfort [14]. The present study validated a simplified approach using an intracoronary hyperemic agent with immediate onset and providing sufficient time for the measurement of IMR. Furthermore, papaverine does not evoke symptoms, and the cost is substantially lower compared to adenosine [16].

The reproducibility of measurement is of utmost importance for its clinical applicability. IMR has been shown to have high intra and interobserver reproducibility. Payne et al. have reported a mean difference between IMR measurements of 0.01 (mean standard error 1.59 (95% CI −3.52 to 3.54)) between observers [19]. In the present study, we observed a mean difference −0.63 (limits of agreements −14.2 to 12.95) between duplicated measurements of IMR using IV adenosine. The reproducibility between repeated measurements using adenosine may be related to its heterogeneous and the unstable effect in the coronary circulation and aortic pressure [20, 21]. Changes in the hyperemic state during the IMR measurement can affect the result affecting its reproducibility. Papaverine, in contrast, provides minimal variation during maximal hyperemia. In this cohort, the mean difference between repeated IMR measurements using IC papaverine was −0.34 (limits of agreements −5.93 to 5.26).

CFR/IMR measurement obtained using intracoronary papaverine was able to reduce in more than 2 minutes the time required to acquire the measurement. The intracoronary route of administration simplified the logistics in the CathLab, avoiding the need to start a peripheral infusion with adenosine. This simplified approach interchanging the hyperemic agent can increase the adoption of microcirculation assessment in clinical practice.

Papaverine has been described as the ideal hyperemic agent [15]. Nonetheless, in approximately 1.4% of the cases, it has been shown to trigger ventricular arrhythmias, namely, torsade de pointes. Okabe et al. have identified multivessel disease as a predictor of torsade de pointes during papaverine administration [22]. In the present study, we used papaverine in patients with no obstructive coronary artery disease and observed no adverse effects. It can be hypothesized that the safety profile of papaverine may be better in patients with no obstructive coronary artery disease. This hypothesis requires further investigation. Additionally, a systematic approach provided by the study environment ensures no residual doses of papaverine were given from an inadequate flush of the guiding catheter or manifold. Dilution of papaverine in a solution that avoided precipitation was also systematically done (Supplementary Figure S4).

The present study has several limitations. First, the relatively small sample size. This, however, was partially circumvented by the acquisition of four IMR measurements per vessel, enabling also to assess test and retest reproducibility. Second, this was a single-center study of physicians trained in the acquisition of bolus thermodilution measurement. The generalizability of these results requires further confirmation. Third, although the protocol of sequential measurements was performed after the return to resting conditions was achieved, we cannot exclude that residual hyperemia was present, thus potentially affecting the subsequent measurement. Fourth, the interchangeability of hyperemic agents proposed in this study is based on comparable results with the traditional technique. We did not evaluate the relationship with patient-related outcomes.

## 5. Conclusion

Assessment of IMR with intracoronary papaverine was feasible and provided similar IMR results to the ones obtained using intravenous adenosine. The use of papaverine as a hyperemic agent led to shorter procedural times, higher reproducibility of IMR, and was more comfortable for the patient. Using intracoronary papaverine for the calculation of IMR simplifies the invasive assessment of the coronary microcirculation in the catheterization laboratory and may increase the adoption of invasive microcirculation resistance measurements.

## Figures and Tables

**Table 1:** Clinical and angiographic characteristics.

*N*, patients	29
Age, years, mean ± SD	68.8 ± 7.24
Gender, male, *n* (%)	18 (62.1)
BMI, mean ± SD	27.8 ± 4.31
Coronary risk factors	
Diabetes, *n* (%)	8 (27.6)
Dyslipidemia, *n* (%)	23 (79.3)
Hypertension, *n* (%)	19 (65.5)
Current smoking, *n* (%)	7 (24.1)
Prior PCI, *n* (%)	7 (24.1)

Clinical presentation	
Stable angina class 1 (%)	1 (3.45)
Stable angina class 2 (%)	5 (17.2)
Stable angina class 3 (%)	1 (3.45)
Stable angina class 4 (%)	1 (3.45)
Unstable angina (%)	1 (3.45)

Silent ischemia	3 (10.3)
Angina equivalent (%)	17 (58.6)
Creatinine clearance, ml/min, mean ± SD	0.82 ± 0.13
Ejection fraction, %, median (95% CI)	60.0 (60.0, 60.0)

Medication	
ACE-I, *n* (%)	13 (44.8)
Beta-blocker, *n* (%)	11 (37.9)
Calcium channel blockers, *n* (%)	4 (13.8)
Statins, *n* (%)	19 (65.5)
Aspirin, *n* (%)	16 (55.2)
P2Y12 inhibitor, *n* (%)	2 (6.9)
Diuretic, *n* (%)	6 (20.7)
Radial access	27 (93%)
Femoral access	2 (7%)

Angiographic characteristics	
Vessel, *n* (%)	
LAD	16 (55.2)
LCX	7 (24.1)
RCA	6 (20.7)
Diameter stenosis, %, median (IQR)	20.0 (14.0, 32.0)
Vessel diameter, mm, mean ± SD	2.60 ± 0.5

BMI, body mass index; PCI, percutaneous coronary intervention; ACE-I, angiotensin-converting enzyme inhibitor; LAD, left anterior descending coronary artery; LCX, left circumflex coronary artery; RCA, right coronary artery.

**Table 2:** Invasive physiologic measurements with adenosine and papaverine.

	Adenosine	Papaverine	*P* value
	*N* = 29	*N* = 29	
IMR, mean ± SD	20.1 ± 8.60	17.7 ± 7.26	0.25
IMR corrected, mean ± SD	19.7 ± 8.51	17.5 ± 7.25	0.28
Tmn during rest, sec, mean ± SD	0.91 ± 0.40	0.92 ± 0.38	0.98
Tmn during hyperemia, sec, mean ± SD	0.29 ± 0.12	0.26 ± 0.11	0.33
Pd, mmHg, mean ± SD	70.7 ± 15.2	69.5 ± 12.5	0.75
Peak temperature, degree, median (IQR)	−4.33 (−5.89, −2.84)	−4.47 (−6.01, −3.75)	0.23
TRT, sec, median (IQR)	0.32 (0.26, 0.48)	0.28 (0.20, 0.36)	0.26
Derivative, degree/sec, median (IQR)	9.95 (5.19, 21.5)	17.3 (8.92, 24.3)	0.16
CFR, mean ± SD	3.15 ± 1.23	3.96 ± 1.58	0.03
FFR, mean ± SD	0.90 ± 0.08	0.92 ± 0.07	0.52
FFR, median (IQR)	0.91 (0.86, 0.97)	0.91 (0.87, 0.98)	0.61

IMR, index of microvascular resistance; IMR corrected, IMR corrected from influence of collateral supply; Tmn, mean transit time; Pd, distal pressure in a coronary; TRT, temperature recovery time; CFR, coronary flow reserve; FFR, fractional flow reserve.

## Data Availability

The data used to support the findings of this study are available from the corresponding author upon request.

## Supplementary Materials

Supplementary Materials Figure S1. Comparison of TMN rest before each CFR/IMR measurement. Figure S2. TMN rest and corrected TIMI frame count. Figure S3. TMN hyperemia and corrected TIMI frame count. Figure S4. Papaverine diluted in pure NaCl 0.9% and papaverine diluted with heparin. Table S1. IMR values in each assessment.
