# Supplementary material for: Simplified Assessment of the Index of Microvascular Resistance
Source: J Interv Cardiol. 2021 Jun 2;2021:9971874. doi: 10.1155/2021/9971874 (PMC8189791; doi:10.1155/2021/9971874)
Supplement: Supplementary Materials — Figure S1. Comparison of TMN rest before each CFR/IMR measurement. Figure S2. TMN rest and corrected TIMI frame count. Figure S3. TMN hyperemia and corrected TIMI frame count. Figure S4. Papaverine diluted in pure NaCl 0.9% and papaverine diluted with heparin. Table S1. IMR values in each assessment. [file 9971874.f1.docx]

*Supplemental Material*

**Simplified Assessment of the Index of Microvascular Resistance**

Monika Kodeboina MD, Sakura Nagumo MD, PhD, Daniel Munhoz MD, PhD, Jeroen Sonck MD, Niya Mileva MD, Emanuele Gallinoro MD, Alessandro Candreva MD, Takuya Mizukami, MD, PhD, Frederik Van Durme MD, Alex Heyse MD, Eric Wyffels MD, Marc Vanderheyden MD, Emanuele Barbato MD, PhD, Jozef Bartunek MD, PhD, Bernard De Bruyne MD, PhD, Carlos Collet MD, PhD^1^

**Running Title:** Intracoronary Papaverine Bolus versus Intravenous Adenosine for IMR assessment

**Address of Correspondence**

Carlos Collet, MD, PhD

Cardiovascular Center Aalst, OLV-Hospital

Moorselbaan 164, 9300 Aalst, Belgium

[carloscollet@gmail.com](mailto:carloscollet@gmail.com) (Carlos Collet)

Tel:+32 53 72 44 39

**Table of contents**

Figure 1S. Comparison of TMN rest before each CFR/IMR measurement ------------------- 3

Figure 2S. TMN rest and Corrected TIMI Frame Count ---------------------------------------- 4

Figure 3S. TMN hyperemia and Corrected TIMI Frame Count -------------------------------- 5

Figure 4S: Papaverine diluted in pure NaCl 0.9% and Papaverine diluted with heparin---- 6

Table S1: IMR values in each assessment----------------- --------------------------------------- 7

**Figure 1S. Comparison of TMN rest before each CFR/IMR measurement**

| 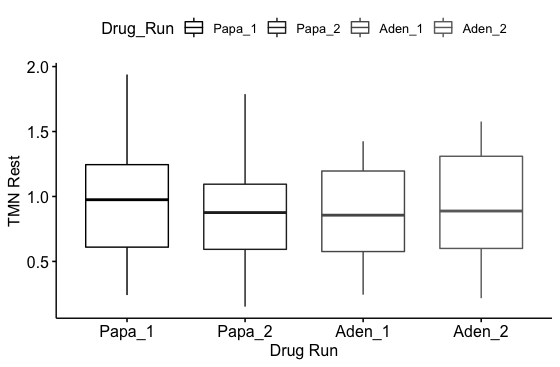    ANOVA: p=0.579 |
| --- |
| ANOVA of the Tmn rest before the first measurement with papaverine (Papa_1), second measurement with papaverine (Papa_2), first measurement with adenosine (Aden_1) and second measurement of adenosine (Aden_2). |
| TMN – Mean transit time; |

**Figure 2S. TMN rest and Corrected TIMI Frame Count**

| 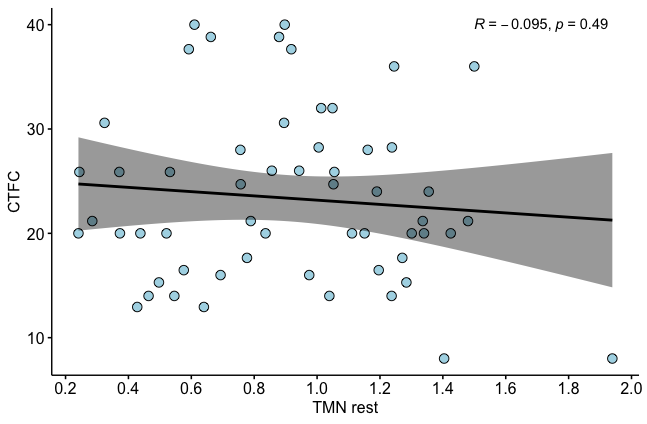 |
| --- |
| Pearson correlation between corrected TIMI Frame count and TMN rest in first measurements with adenosine and papaverine; |
| TMN – Mean transit time; CTFC – corrected TIMI frame count; |

**Figure 3S. TMN hyperemia and Corrected TIMI Frame Count with Papaverine and Adenosine**

| A **Papaverine B** **Adenosine**    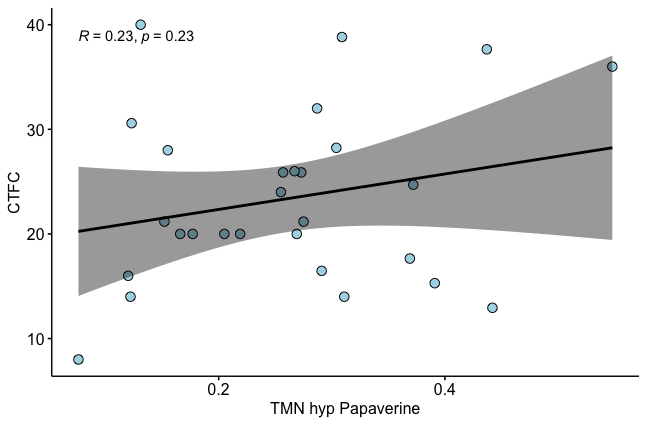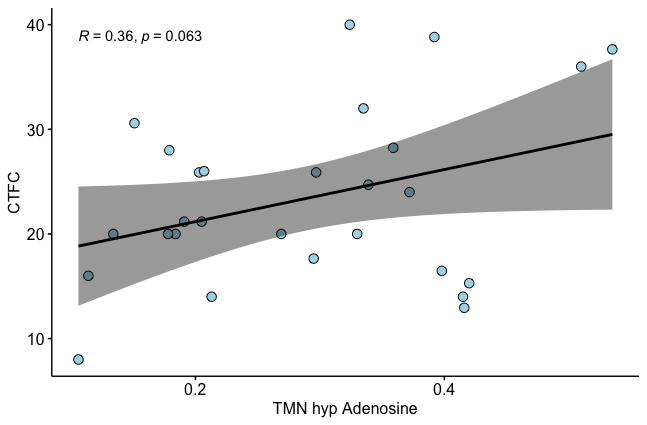 |
| --- |
| Pearson correlation between corrected TIMI Frame count and TMN hyperemia in first measurements with A: Papaverine and B: Adenosine; |
| TMN – Mean transit time; CTFC – corrected TIMI frame count; hyp – hyperemia; |

**Figure 4S: Papaverine diluted in pure NaCl 0.9% and Papaverine diluted with heparin.**

A: Transparent solution of papaverine diluted with only NaCl 0.9%. B: Milky solution of papaverine diluted with NaCl 0.9% and heparin

**Table 1S: IMR values in each assessment**

| Record Id | IMR Papaverine 1^st^ measurement | IMR Adenosine  1^st^ measurement | IMR Papaverine 2^nd^ measurement | IMR Adenosine2^nd^ measurement |
| --- | --- | --- | --- | --- |
| 1 | 24 | 24 | 24 | 21 |
| 2 | 14 | 16 | 14 | 17 |
| 3 | 22 | 34 | 29 | 34 |
| 4 | 15 | 17 | 14 | 14 |
| 5 | 8 | 9 | 14 | 11 |
| 6 | 13 | 14 | 16 | 22 |
| 7 | 30 | 37 | 27 | 32 |
| 8 | 8 | 7 | 9 | 6 |
| 9 | 7 | 9 | 9 | 9 |
| 10 | 13 | 17 | 14 | 16 |
| 11 | 12 | 12 | 9 | 8 |
| 12 | 34 | 34 | 33 | 38 |
| 13 | 10 | 36 | 9 | 65 |
| 15 | 15 | 15 | 14 | 15 |
| 16 | 14 | 11 | 13 | 9 |
| 17 | 26 | 30 | NA | NA |
| 18 | 21 | 16 | 22 | 23 |
| 19 | 22 | 17 | 24 | 19 |
| 20 | 17 | 18 | 14 | 15 |
| 21 | 8 | 12 | 6 | 9 |
| 22 | 27 | 28 | 26 | 30 |
| 23 | 27 | 31 | 27 | 32 |
| 24 | 17 | 22 | 23 | 27 |
| 25 | 18 | 24 | 18 | 24 |
| 26 | 18 | 14 | 15 | 18 |
| 27 | 14 | 14 | 11 | 13 |
| 28 | 23 | 24 | 27 | 24 |
| 29 | 23 | 19 | 24 | 22 |
